# Poisson Parameters of Antimicrobial Activity: A Quantitative Structure-Activity Approach

**DOI:** 10.3390/ijms13045207

**Published:** 2012-04-24

**Authors:** Radu E. Sestraş, Lorentz Jäntschi, Sorana D. Bolboacă

**Affiliations:** 1 University of Agricultural Science and Veterinary Medicine Cluj-Napoca, 3-5 Mănăştur, Cluj-Napoca 400372, Romania; E-Mail: rsestras@usamvcluj.ro; 2 Technical University of Cluj-Napoca, 28 Memorandumului, Cluj-Napoca 400114, Romania; 3 Department of Medical Informatics and Biostatistics, “Iuliu Haţieganu” University of Medicine and Pharmacy Cluj-Napoca, 6 Louis Pasteur, Cluj-Napoca 400349, Cluj, Romania; E-Mail: sbolboaca@umfcluj.ro

**Keywords:** oils compounds, antimicrobial effect, bacteria and fungi species, probability distribution function, quantitative structure-activity relationship (QSAR), multiple linear regression (MLR)

## Abstract

A contingency of observed antimicrobial activities measured for several compounds *vs*. a series of bacteria was analyzed. A factor analysis revealed the existence of a certain probability distribution function of the antimicrobial activity. A quantitative structure-activity relationship analysis for the overall antimicrobial ability was conducted using the population statistics associated with identified probability distribution function. The antimicrobial activity proved to follow the Poisson distribution if just one factor varies (such as chemical compound or bacteria). The Poisson parameter estimating antimicrobial effect, giving both mean and variance of the antimicrobial activity, was used to develop structure-activity models describing the effect of compounds on bacteria and fungi species. Two approaches were employed to obtain the models, and for every approach, a model was selected, further investigated and found to be statistically significant. The best predictive model for antimicrobial effect on bacteria and fungi species was identified using graphical representation of observed *vs*. calculated values as well as several predictive power parameters.

## 1. Introduction

Plant extracts, including oils, have been used as therapeutics from ancient times and have been reinvented more often in the last years. Important medical effects of plant extracts have been identified during the time (antioxidant, antimicrobial [1–4]) and some mechanisms of actions were investigated [5–8]. Research on plant extracts on specific symptoms and diseases is carried out all over the world [9–11]. New approaches are applied in drug industry in order to identify promising medicinal plant as source of new drugs and drug leads [12] even if pharmaceutical companies significantly decreased their activities in natural product discovery during the past few decades [13].

Quantitative Structure-Activity Relationships (QSARs) are mathematical models resulting from the application of different statistical approaches in correlation analyses of biologic activity and/or physical or chemical properties of active compounds with descriptors derived from structure and/or properties [14]. Traditional strategies based on animal models are nowadays replaced by *in silico* approaches by moving the experiments into virtual laboratories [15,16]. These *in silico* approaches are sustained by the increased power of computers and are widely used due to low costs (no costs for compounds synthesize), possibility to investigate not synthesized compounds as well as possibility to investigate huge amount of promising chemicals. Different QSAR approaches demonstrated their effectiveness in drug design [17,18] and in screening of active compounds [19,20], also with regards to natural products [21,22]. Several methods like MARCH-INSIDE [23,24], TOPS-MODE [25], and TOMO-COMD [26] have been used in QSAR investigation of anti-bacterial drugs [27,28] (including anti-fungi [29], anti-parasite [30], and anti-viral drugs [31]). The MARCH-INSIDE method was further integrated in the Bio-AIMS online platform and can be used as a prediction tool for new anti-microbial drugs or their protein targets [32].

Jirovetz *et al*. investigated the antimicrobial effects of a series of oils components, oils and mixtures on gram-positive and -negative bacteria (*Staphylococcus aureus*, *Enterococcus faecalis*, *Escherichia coli*, *Pseudomonas aeruginosa*, *Klebsiella pneumoniae*, *Proteus vulgaris*, *Salmonella* sp.) and *Candida albicans* [33]. In the present research we focused on two major objectives based on the experimental observations of Jirovetz *et al*. [33]. The first objective was to identify the probability distribution function of the antimicrobial effects of compounds, oils and mixtures on above-presented bacteria and fungus species. Identification of the probability distribution function allows us to compute the population parameters, an overall estimator of the antimicrobial effect that comprises the antimicrobial potencies on different species in a single value. The second objective was to find the appropriate predictivity measures of quantitative structure-activity relationship using the context of the overall antimicrobial activity of 22 active compounds.

## 2. Results

### 2.1. Probability Distribution Analysis

**Figure 1:**
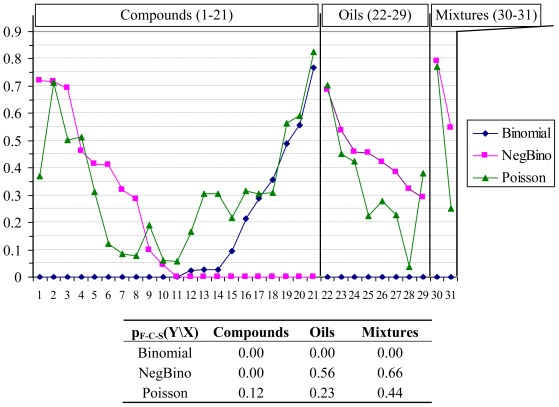
Results of probability distribution functions analysis. X: Compounds (**1**–**21; 1** = Citral, **2** = Geraniol, **3** = Geranyl formate, **4** = Geranyl acetate, **5** = Geranyl butyrate, **6** = Geranyl tiglate, **7** = Neral, **8** = Nerol, **9** = Nerol acetate, **10** = Neryl butyrate, **11** = Neryl propanoate, **12** = Citronellal, **13** = Citronellyl formate, **14** = Citronellyl acetate, **15** = Citronellyl butyrate, **16** = Citronellyl isobutyrate, **17** = Citronellyl propionate, **18** = Hydroxycitronellal, **19** = Rose oxide, **20** = Eugenol, **21** = Sulfametrole, **32** = Citronellol), Oils (**22**–**29; 22** = Citronella, **23** = Geranium Africa, **24** = Geranium Bourbon, **25** = Geranium China, **26** = Helichrysum, **27** = Palmarosa, **28** = Rose, **29** = Verbena), Mixtures (**30**–**31; 30** = Tetracycline hydrochloride, **31** = Ciproxin); Y: Binomial (◆), NegBino (■), Poisson (▴); “Is Y the distribution of any X on bacteria and fungi species?”.

The antimicrobial effects at contingency of compounds, oils and mixtures on bacteria were investigated to identify the probability distribution function along bacteria series. The Uniform distribution was rejected at the beginning of the analysis due to unreasonable estimates of the population parameters. The remained three discrete distributions were compared based on several agreements. The percentage of rejection according to Fisher’s Chi-Square global statistics for each identified probability distribution function according to the class (as compounds, oils, mixtures) is shown in Figure 1 (detailed data can be found in Supplementary material). The following null hypothesis was tested using F-C-S statistic (F-C-S values in Figure 1): “The parameters of the identified distribution follow for each series of compound/oil/mixture the Binomial/NegBinomial/ Poisson distribution”.

Statistical parameters and estimates of the population properties under assumption of Poisson distribution are presented in Table 1.

Assuming the Poisson distribution (as the F-C-S value from Figure 1 allowed us to do), statistical parameter (λ) and population properties were computed for Citronellol (CID = 8842, with less than 5 observations, not included in verification of the Poisson distribution assumption-see Supplementary material) and the following results were obtained: λ = 14.5, Mode = 14, Mean = 14.500, Variance = 4.500, Standard Deviation = 3.808, Skewness = 0.263, Excess Kurtosis = 0.069, Median = 13.832.

### 2.2. QSAR Models

Two requirements were imposed in identification of the proper transformation of Poisson parameter λ: the absence of outliers and the presence of normality at a significance level of 5%. The global F-C-S distribution statistic indicated that the Poisson parameter more likely follows a Log-normal distribution (statistics: K–S = 0.1315; p_K–S_ = 0.7948; A–D = 0.3874; Crit_A–D5%_ = 2.5018 (critical values associated for Anderson-Darling test); C–S_df = 2_ = 0.9403; p_C–S_ = 0.6249).

The Eugenol compound was identified as outlier with Grubbs’ test (Z = 3.178, Z_critical–5%_ = 2.7338). After natural logarithm transformation of the Poisson parameters, seen as an overall antimicrobial activity of investigated compounds, no other outlier was identified (the highest Z value was of 2.528; Z_critical–5%_ = 2.758) and the normality hypothesis of the ln(λ) values could not be rejected (*p* > 0.05). Further testing on ln(λ) under the normal distribution assumption gave no reason to reject the normality of the data in the training test (K–S = 0.14351, p_K–S_ = 0.917; A–D = 0.37751, p_A–D_ = 0.686; C–S = 0.62246, p_C–S_ = 0.430; F–C–S = 1.307; p_F–C–S_ = 0.727) nor in test set (K–S = 0.2301, p_K–S_ = 0.779; A–D = 0.3860, p_A–D_ = 0.679; F–C–S = 0.637; p_F–C–S_ = 0.727).

#### 2.2.1. Based on DRAGON Descriptors

**Figure 2:**
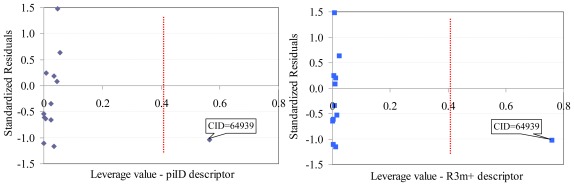
Williams plot (training set): Dragon descriptors.

Sulfametrole (CID = 64939) proved to be influential in the model obtained based on Dragon descriptors (training set, Figure 2). Both Dragon descriptors proved to be higher than expected (h_i–piID_ = 0.5643, h_i–R3m+_ = 0.7602, where piID and R3m+ are Dragon descriptors) for Sulfametrole compound.

The overall correlation between Dragon descriptors obtained for whole data set (*n* = 21 compounds) was of 0.8461 (*p* < 0.0001). Moreover, a statistically significant correlation was obtained between ln(λ) and R3m+ descriptor (*r* = 0.4800, *p* = 0.0220).

The results of regression analysis with Dragon descriptors provided the equation presented in Equation(1) relating ln(λ) with compounds structure, after the withdrawal of Sulfametrole from the training set.

(1) $$\begin{array}{l} \hat{\text{Y}}=3.626(\pm 0.496)-0.045(\pm 0.012)\cdot \text{piID}+18.569(\pm 19.404)\cdot \text{R}3\text{m}+\\ n_{\text{TR}}=12;{R^{2}}_{\text{TR}}=0.8970;{R^{2}}_{\text{Adj}-\text{TR}}=0.8741;F_{\text{TR}}(p)=39\;(3.62\times 10^{-5});{\text{se}}_{\text{TR}}=0.1037;\\ p_{\text{intercept}}=4.86\times 10^{-8};p_{\text{piID}}=1.28\times 10^{-5};p_{\text{R}3\text{m}+}=0.058;\\ T_{\text{piID}}=T_{\text{R}3\text{m}+}=0.776;{\text{VIF}}_{\text{piID}}={\text{VIF}}_{\text{R}3\text{m}+}=1.305;\\ R_{(\text{Y}-\text{piID})\text{TR}}=-0.9183\;(p-\text{value}=2.50\times 10^{-5});R_{(\text{Y}-\text{R}3\text{m}+)\text{TR}}=-0.2410\;(p-\text{value}=0.4505);\\ R_{(\text{piID}-\text{R}3\text{m}+)\text{TR}}=0.4833\;(p-\text{value}=0.1114);\\ {R^{2}}_{\text{loo}}=0.8452;F_{\text{loo}}\;(p)=24(2.35\times 10^{-4});{\text{se}}_{\text{loo}}=0.1276;\\ n_{\text{TS}}=7;{R^{2}}_{\text{TS}}=0.6518;F_{\text{TS}}(p)=11\;(2.16\times 10^{-2});\\ R_{(\text{Y}-\text{piID})\text{TS}}=-0.0869\;(p-\text{value}=0.8241);R_{(\text{Y}-\text{R}3\text{m}+)\text{TS}}=-0.2410\;(p-\text{value}=0.0024);\\ R_{(\text{piID}-\text{R}3\text{m}+)\text{TS}}=0.3469\;(p-\text{value}=0.3604) \end{array}$$

where Ŷ = ln(λ) estimated by Equation(1); *R*^2^ = determination coefficient; TR = training set; loo = leave-one-out analysis; TS = test set; *Ext* = external set; *R*^2^_Adj_ = adjusted determination coefficient; *F* = *F*-value (from ANOVA table); *p* = *p*-value associated to *F*-value; *se* = standard error of estimate; Dragon descriptors: piID = conventional bond order ID number-walk and path counts; R3m+ = R maximal autocorrelation of lag 3/weighted by mass GETAWAY descriptors; *T* = Tolerance; VIF = Variance Inflation Factor; *R* = correlation coefficient.

**Figure 3:**
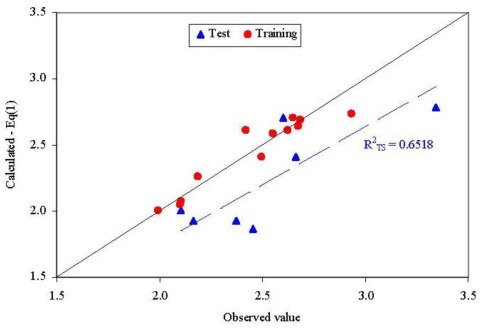
Observed *vs*. calculated parameter: QSAR-Dragon (Equation (1)*R*^2^_TS_ = determination coefficient in test set).

The abilities in estimation (training set) and prediction (test set) of the model from Equation(1) are presented in Figure 3. No statistically significant difference could be identified when the goodness-of-fit was compared in training set and test set for the model presented in Equation (1) (*Z* = 0.3590, *p* = 0.3598).

#### 2.2.2. Based on SAPF Descriptors

**Figure 4:**
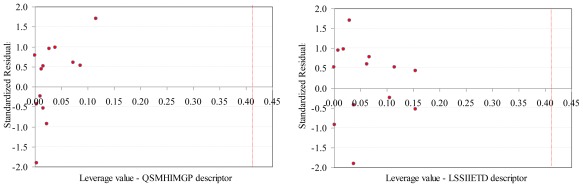
Williams plots (training set): SAPF descriptors.

No leverage was identified when the SAPF descriptors were investigated (Figure 4).

The overall correlation between SAPF descriptors obtained for whole data set (*n* = 22 compounds) was of 0.4800 (*p* = 0.0238). Moreover, a statistically significant correlation was obtained between ln(λ) and LSSIIETD descriptor (*r* = −0.5249, *p* = 0.0122).

**Figure 5:**
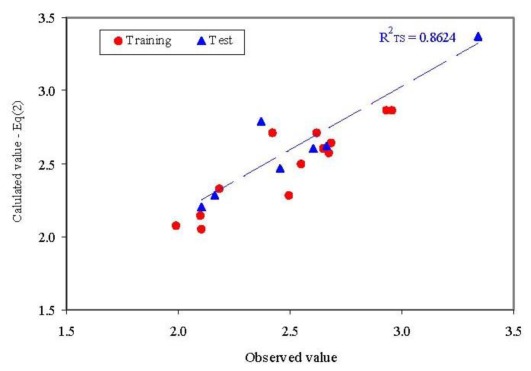
Observed *vs*. calculated parameter: QSAR-SAPF (Equation (2)*R*^2^_TS_ = determination coefficient in test set).

The results of regression analysis with SAPF descriptors relating ln(λ) with compounds structure by using the entire training set is presented in Equation(2).

(2) $$\begin{array}{l} \hat{\text{Y}}=3.858(\pm 0.502)+0.398(\pm 0.189)\cdot \text{QSMHIMGP}-0.149(\pm 0.048)\cdot \text{LSSIIETD}\\ n_{\text{TR}}=13;{R^{2}}_{\text{TR}}=0.8286;{R^{2}}_{\text{Adj}-\text{TR}}=0.7944;F_{\text{TR}}(p)=24\;(1.48\times 10^{-4});{\text{se}}_{\text{TR}}=0.1419;\\ p_{\text{intercept}}=9.66\times 10^{-9};p_{\text{QSMHIMGP}}=8.37\times 10^{-4};p_{\text{LSSIIETD}}=3.93\times 10^{-5};\\ R_{(\text{Y}-\text{QSMHIMGP})\text{TR}}=-0.0122\;(p-\text{value}=0.9684);\;\;\;R_{(\text{Y}-\text{LSSIIETD})\text{TR}}=-0.6705\\ \;(p-\text{value}=0.0121);\;R_{(\text{QSMHIMGP}-\text{LSSIIETD})\text{TR}}=06862\;(p-\text{value}=0.0096);\\ T_{\text{QSMHIMGP}}=T_{\text{LSSIIETD}}=0.529;{\text{VIF}}_{\text{QSMHIMGP}}={\text{VIF}}_{\text{LSSIIETD}}=1.890;\\ {R^{2}}_{\text{loo}}=0.6998;F_{\text{loo}}\;(p)=11(2.90\times 10^{-3});{\text{se}}_{\text{loo}}=0.1910;\\ n_{\text{TS}}=7;{R^{2}}_{\text{TS}}=0.8624;F_{\text{TS}}(p)=24\;(4.41\times 10^{-3});\\ R_{\text{(Y}-\text{QSMHIMGP)TS}}=0.7511\;\;\;(p-\text{value}=0.0516);\;\;\;R_{\text{(Y}-\text{LSSIIETD)TS}}=-0.3725\\ \;(p-\text{value}=0.4106);R_{(\text{QSMHIMGP}-\text{LSSIIETD})\text{TS}}=0.2250\;(p-\text{value}=0.6276) \end{array}$$

where Ŷ = ln(λ) estimated/predicted by Equation (2); *R*^2^ = determination coefficient; *R* = correlation coefficient; TR = training set; loo = leave-one-out analysis; TS = test set; *R*^2^_Adj_ = adjusted determination coefficient; *F* = *F*-value (from ANOVA table); *p* = *p*-value associated to *F*-value; se = standard error of estimate; QSMHIMGP and LSSIIETD = SAPF descriptors; *T* = tolerance; VIP = Variance Inflation Factor. The abilities in estimation (training set) and prediction (test set) of the model from Equation (2) are presented in Figure 5.

No statistically significant difference was identified when the goodness-of-fit in training and test sets were compared for the model presented in Equation (2) (Z-statistics = 0.3590, p = 0.3598).

The search for the best fit between observed and linear regression model with two descriptors when the joined pool of SAPF and Dragon descriptors retrieved the same model as the one from Equation (2).

#### 2.2.3. Models Comparison

Parameters defined in Material and Method section were used to compare the QSAR-Dragon model with QSAR-SAPF model. The residuals, defined as the difference between observed value and calculated value based on identified models, are presented in Table 2. The values of the parameters used in models assessment analysis were presented in Table 3.

Two compounds were randomly chosen as external set. The predictions that were closest to the observed values were obtained by QSAR-SAPF model (Equation (2); Table 2).

Steiger’s test was used to identify if there are any statistically significant differences in terms of correlation coefficient between the models from Equation (1) and the model from Equation (2). The lowest *p*-value was obtained when the correlation coefficient in training sets was compared (*Z*-statistics = −1.4511, *p* = 0.0734). This suggests that the models are close to being statistically different.

## 3. Discussion

The antimicrobial effects of chemical compounds on bacteria and fungi species were analyzed with regards to probability distribution function. In addition, a structure-activity relationship analysis able to describe the effect of chemical compounds on the entire population of bacteria and fungi species was successfully conducted.

The analysis of Figure 1 revealed that for compounds series there is at least one sample with no fit (0.00 probability of agreement) for both Binomial and Negative Binomial distributions. Poisson distribution always had the probability of agreement above 0.05 (the hypothesis of Poisson distribution cannot be rejected at 5% significance level), being the only discrete distribution from investigated ones that showed this behavior. Furthermore, the p_F-C-S_ value provided a global agreement of 12% for “Is Poisson the distribution of any compound on bacteria and fungi species?”, enough to assure us that the Poisson distribution is the true distribution of compounds’ antimicrobial activities on the studied bacteria and fungi species. The situation is somehow reversed for oils and mixtures; if the Poisson distribution is the only one not rejected for compounds, then the Negative Binomial distribution also cannot be rejected for oils and mixtures. A deeper investigation on factors influencing antimicrobial activities may reveal that the negative binomial distribution should be rejected for the whole data presented in Table 4. The reason for this fact should be foundd in the distribution of the compounds series activities on a given bacteria (columns data in Table 4).

Thus, it was already proven [34] that Negative Binomial distribution occurs when both column and row data are shaped by Poisson distribution, which is not our case since only rows (a compound activity) are shaped by Poisson distribution (see Figure 1). Moreover, rows data from Table 4 are more likely to be Negative Binomial distributed, suggesting that at least two factors coexist in the compounds’ structure and influence their activity.

The analysis of distribution on bacteria and fungi species revealed the following:

- Compounds series:
  ○ Without any exception, the antimicrobial effects of all investigated compounds proved to follow Poisson distribution. Moreover, the hypothesis that any compound has a Poisson distribution of antimicrobial activity on bacteria population could not be rejected by F-C-S statistics (F-C-S statistics = 28.79, *p* = 0.12, Figure 1). Starting with this result, the Poisson λ parameter has been obtained to reflect what happen in the population, this parameter being an estimate for both central tendency and variability of antibacterial effects. The analysis of the obtained Poisson parameters showed to follow more likely a log-normal distribution and a logarithm transformation was applied on these values before quantitative structure-activity relationship search. This transformation was applied to avoid the presence of outliers and to assure the normality assumption needed for linear regression analysis [35,36].
  ○ Negative binomial distribution was rejected by 55% of compounds while Binomial distribution was rejected in 70% of cases. Negative binomial distribution, also known as the Pascal distribution or Pólya distribution, is a twin of Poisson distribution [37,38] widely used in analysis of count data [39,40]. The negative binomial distribution could be obtained by superposition of a continuous distribution over Poisson distribution (Fisher showed the convolution between Chi-Square and Poisson distribution [41]). Other authors showed that the negative binomial distribution might derive from a convolution between the Gamma distribution (Chi-Square distribution is a particular case of Gamma distribution) and Poisson distribution [42,43]. Whenever the separation of factors is possible, it is also possible to separate the convolutions of distributions [44], and this separation give the possibility to analyze separately the factors. The results presented by Jäntschi *et al*. [44] sustained and/or are sustained by convolution of Poisson distribution with a continuous distribution in regards of both factors (bacteria and chemical compounds) in the expression of antimicrobial activity. The results showed that antimicrobial activity follow a negative binomial distribution under the influence of both factors (bacteria and chemical compound) and Poison distribution under the influence of the bacteria factor [44]. Furthermore, the negative binomial distribution might be obtained by convolution of log-normal with Gamma distribution; although a high number of observations are needed (*n* > 250) in order to statistically assure the difference between Log-normal and Gamma distributions [45].
- Oils and mixture series:
  ○ Negative Binomial distribution cannot be rejected for oils. Moreover, Negative Binomial distribution for oils had a higher likelihood than Poisson distribution (*p*_F-C-S_ for Negative Binomial: 0.56; *p*_F-C-S_ for Poisson: 0.23) while the Binomial distribution was rejected.
  ○ Negative Binomial distribution cannot be rejected for mixtures either. Moreover, Negative Binomial distribution for mixtures had also higher likelihood than Poisson distribution (*p*_F-C-S_ for Negative Binomial = 0.66; *p*_F-C-S_ for Poisson = 0.44) while the Binomial distribution was rejected.
  ○ The above-presented facts suggest that in the case of oils and mixtures, the factors of the antibacterial activity are not completely separated when oil/mixture name are taken as factor; this appears to be because the Negative Binomial distribution often occurs when a convolution/superposition of Poisson distributions characterize the observed data [46].

Overall, any investigated compound, oil and mixture proved to have an antimicrobial effect that follows the Poisson distribution on studied bacteria and fungi species. The λ Poisson parameter, varied from 7.286 (Nerol acetate) to 28.250 (Eugenol) and represents the mean and variance of inhibition zone of compound/oil/mixture on investigated species. The obtained parameter of Poisson distribution proved able to characterize the overall antimicrobial activity (both mean and variance equals to Poisson parameter λ, Table 1) of the compounds on the investigated bacteria population.

The structure-activity relationships between compounds’ structure and the overall antimicrobial effect on bacteria population, as well as the suitability of a pool of descriptors (SAPF and Dragon approaches) for the overall antimicrobial activity estimation and prediction were furthermore investigated.

QSAR model with two descriptors that proved abilities in estimation and prediction was identified for each approach after the split of compounds in training (13 compounds), test (7 compounds) and external (2 compounds) sets. Normal distribution of the observations was assured through natural logarithm transformation (*p* > 0.05) to allow investigation of structure (of compounds)-activity (overall antimicrobial activity) relationships using multiple linear regression.

The analysis of QSAR-Dragon model revealed the following:

- One compound proved to be influential in the model (CID = 64939, Figure 2). This compound obtained the value of leverage for both Dragon descriptors higher than the accepted threshold (0.41). This compound, which belongs to the training set, was withdrawn, and a model based on 12 compounds in training set was obtained, Equation(1).
- Two descriptors were able to describe the linear relation between overall antimicrobial activities of investigated compounds. One descriptor belongs to the walk and path counts and relates the conventional bond order ID number while the second descriptor relates the maximal autocorrelation of lag 3 divided by mass (R3m+). According with associated coefficients, the R3m+ had a higher contribution in the model compared with piID descriptor, but its contribution is to the significance level threshold (5.8% compared to imposed 5% significance level).
- QSAR-Dragon model proved to be statistically significant (*F* = 39, *p* = 3.62 × 10^−5^). A low value of root mean square error was obtained in leave-one-out analysis (0.1276). The contribution of R3m+ descriptor to the model is questionable since the significance associated to its coefficient is very close to 0.05 but since it has a real contribution in the *r*^2^ value its significance of 5.8% was accepted. Moreover, the R3m+ proved not significantly correlate with Poisson parameter (*r* = −0.2410).
- Multicollianearity is not present in the model since the tolerance value 0.1 < *T* < 1 and the variance inflation factors (VIF) < 10 even if a significant correlation coefficient was obtained between Dragon descriptors.
- The model proved its abilities in estimation (*R*^2^_TR_ = 0.897) as well as in prediction (internal validity of the model in leave-one-out analysis, *R*^2^_loo_ = 0.845 and external validation in test set *R*^2^_TS_ = 0.652) with a difference in the goodness-of-fit from 0.052 (training *vs*. interval validation - leave one out analysis) to 0.245 (training *vs*. external validation-test set). However, the difference of 0.245 proved not statistically significant (*p* > 0.05).
- Unfortunately, external abilities in prediction were away from the expected abilities. The trend is significant far from the expected line-Figure 3.
- The abilities in estimation (training set) proved not statistically significant from the abilities in prediction (test set) since a probability of 0.3598 was obtained in comparison.

The analysis of QSAR-SAPF model revealed the following:

- The values of SAPF descriptors associated to compounds proved that no compound had significant influence on the model (all leverage values where lower than threshold −0.41, Figure 4).
- SAPF model proved statistically significant (*F* = 24, *p* = 1.48 × 10^−4^). The contribution of both descriptors to the model proved statistically significant (*p*-values associated to coefficients <0.05).
- According to descriptors from Equation(2), the global model of antibacterial activity is related to both molecular geometry and topology: one descriptor identified a relation between the geometry of compounds and the overall antimicrobial activity while the second descriptor identified a relation with compounds’ topology. Moreover, the atomic mass and electronegativity proved to be related to the overall antimicrobial activity by the same split ratio in the expression of the model descriptors.
- Multicollianearity was not identified in the QSAR-SAPF model, even if a statistically significant correlation coefficient between descriptors exists (the tolerance values were higher than 0.1 and smaller than 1 and the variance inflation factors (VIF) had values smaller than 10).
- The model proved its abilities in estimation (*R*^2^_TR_ = 0.829) as well as in prediction (internal validity of the model in leave-one-out analysis, *R*^2^_loo_ = 0.700 and external validation in test set *R*^2^_TS_ = 0.862) with a difference in the goodness-of-fit from −0.034 (training *vs*. external validation - test set) to 0.129 (training *vs*. interval validation-leave one out analysis). Moreover, none of these differences were statistically significant (*p* > 0.05).
- External abilities in prediction proved to be close to expected abilities for QSAR-SAPF model (Figure 5).

The comparison of the identified models revealed the following:

- Dragon model has slightly better abilities in estimation compared to SAPF model, but these abilities proved not statistically significant. The determination coefficient obtained both in training set and in leave-one-out analysis was higher compared to SAPF model with 0.068 and respectively 0.145. Moreover, the abilities of prediction seem to be better for SAPF model compared to Dragon model (a difference of 0.211, not statistically significant *p* < 0.05). This observation is also sustained by the lowest value of residuals in training set for Dragon model and in two compounds from training set and all compounds from test set for SAPF model (Table 2).
- The SAPF model systematically obtained smallest values of parameters presented in Table 3: best explaining the variability in the observation; smallest typical errors; smallest standard error of prediction as well as smallest relative error of prediction. The highest difference is observed with regards to standard error of prediction that is almost 4 times higher for Dragon model compared to SAPF model.
- The analysis of predictive power of the models demonstrated that SAPF model had significantly higher power of prediction (Table 3). According to the obtained results, the *Q*^2^ values for Dragon model are smaller than 0.6, being considered unacceptable while all *Q*^2^ values for SAPF model are higher than 0.77. These results show that the Dragon model can be rejected from a statistical point of view, taking also into consideration that the relative error of prediction is almost 2 times higher compared to SAPF model.
- Furthermore, the mean of residuals for training, external and external + test set proved not statistically different by zero when the SAPF model was analyzed. The Fisher’s predictive power identified statistically difference by zero of the residuals obtained by Dragon model in both training and test sets (9 compounds) (*p* < 0.05, Table 3).
- The model with a higher concordance between observed and estimated/predicted could be considered the best model. The analysis of concordance correlation coefficient revealed a substantial strength of agreement for training set but a very poor agreement in test set for Dragon model. A moderate strength of agreement was obtained by SAPF model in both training and test sets (Table 3).
- Steiger’s test was not able to identify any statistically significant differences between Dragon and SAPF model regarding goodness-of-fit neither in training set nor in external set.

It can be concluded based on the facts presented above that the SAPF model is a reliable, valid (internally as well as externally) and stable model useful in characterization of overall antimicrobial activity on investigated compounds, both in terms of estimation and prediction.

The aim and objectives of the research have been achieved. The antimicrobial effect proved to follow the Poisson distribution and its parameter was furthermore used to identify those descriptors from Dragon and SAPF pools able to characterize the link between compounds and overall antimicrobial activity. Two newly developed models were found statistically valid. However, which of these QSAR models is better? The analysis of applicability domain of the models obtained in training sets was able to identify based on the values of descriptors one structurally influential compound in training set for Dragon model. According to the obtained results, one compound was withdrawn from further analysis in Dragon modeling. Dragon model was created based on 12 compounds in training set while the SAPF model was created based on 13 compounds in training set. Graphical representation of observed *vs*. calculated values based on identified models as well as the predictive power parameters showed that the best model to be applied on new chemicals is the SAPF model.

## 4. Experimental Section

### 4.1. Compounds, Oils and Mixtures

The antimicrobial effects of twenty-two compounds, eight oils and two mixtures on gram-positive and -negative bacteria (*Staphylococcus aureus*, *Enterococcus faecalis*, *Escherichia coli*, *Pseudomonas aeruginosa*, *Klebsiella pneumoniae*, *Proteus vulgaris*, *Salmonella* sp.) and on one fungus (*Candida albicans*), expressed as inhibition zone (mm, Agar diffusion disc method [33]), were included in the analysis (Table 4). The PubChem database was used to retrieve the compounds structure and associated CIDs (Compound IDentification numbers); the data are presented in Table 4.

### 4.2. Distribution Analysis

Since all inhibition zones expressed in mm are integer numbers, a search for a discrete distribution was conducted having as alternatives Uniform, Binomial, Negative Binomial and Poisson distributions (other alternatives were excluded due to lack of fit with observed data). Kolmogorov-Smirnov (K-S) [47] and Anderson-Darling (A-D) [48] statistics were used to measure the departure between observations and a certain probability distribution function (PDF). Fisher’s method combining independent tests for significance (Fisher’s Chi-Square, abbreviated as F-C-S [49]) was used to obtain a global probability of agreement between the distribution and the observed samples.

The whole pool (matrix) of data was prior analyzed and none of the above distribution functions give an acceptable (higher than 5%) agreement with the observations. This fact could be explained by the heterogeneity of the chemicals/oils/mixtures.

In order to obtain the PDF of antimicrobial effects of compounds, oils and mixtures on bacteria and fungus population, rows of experimental values were analyzed as independent samples. A number of five observations in sample qualified the sample for estimation of the distribution parameters, and the analysis was conducted using maximum likelihood estimation (MLE) [34] procedure. The measure of the agreement was expressed using the probability of F-C-S test. Also the following hypothesis was tested: a certain PDF can be accepted for populations of all samples regardless of PDF parameters values. The identified PDF was further used to estimate the population parameter(s) for sample(s) without enough data (e.g., Citronellol, see Table 4).

Population statistics of the identified PDF can be seen as an estimator of overall antimicrobial activity of the investigated compound on the bacteria and fungi population. The series of the population statistics for all investigated compounds was furthermore subject of a structure-activity relationship search intended to relate the overall antimicrobial effect with compounds’ structure.

### 4.3. Molecular Descriptors Calculation

The molecular modeling study was conducted at PM3 semi-empirical level of theory [50] on chemical compounds series.

A series of home-made programs were used to perform the following tasks: ■ automate transformation the *.sdf or *.mol files as *.hin files; ■ prepare the compounds for modeling (run HyperChem v.8.0 [51] with HyperChem scripts in order to obtain molecular models) [52]; ■ calculate the molecular descriptors (SAPF approach) for all compounds (calculate all descriptors; select a relevant subset of descriptors); ■ split the set randomly in training (for model development, ~2/3 compounds in training set) and two test sets (for model validation); ■ search for multiple linear regression (search for two descriptors linear models) in training set; ■ validate the model obtained in training set on test sets.

**Figure 6:**
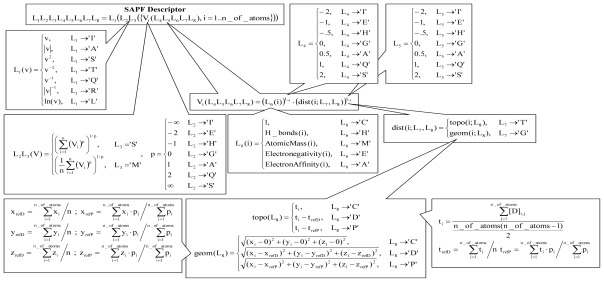
SAPF descriptors (v = value, ln = natural logarithm, V = vector, T = topology, G = geometry, x, y, z = geometric atomic coordinates, i = atom, refD = modality to calculate coordinates—from average, refP = modality to calculate coordinates—from property center formula, t = topological atomic coordinate.

The molecular descriptors for the chemical compounds were calculated using a home-made software that implemented Structural Atomic Property Family [53,54] (SAPF approach, methodology of calculation depicted in Figure 6) and the Dragon software [55] (all Dragon descriptors).

The SAPF approach is a method that cumulates atomic properties at the molecular level. The approach used a localization of the molecular center using a metric, an atomic property (C = cardinality (number of heavy atoms), H = Hydrogen bonds (number of Hydrogen atoms), M = atomic mass (relative units), E = electronegativity (on Pauling scale [56]), and A = electron affinity), a power of a distance as well as of an atomic property in the expression of descriptor in regard to atomic effect, a modality of accumulation of atomic properties at the molecular level, and a linearization operation (see Figure 6).

### 4.4. Identification and Characterization of Linear Regression Models

Linear regression models (additive models) were used for search of structure-activity relationship between overall antimicrobial effects as dependent variable and structural descriptors (from SAPF approach and Dragon software) as independent variables.

Kolmogorov-Smirnov, Anderson-Darling, and Chi-Square statistics [57] as well as Grubbs test for outliers [58] were used to decide which transformation should be applied to assure the normality of observations (in our case the parameter of the probability distribution function) [50,51].

Regression analysis was employed to select the candidate models and the following criteria were used: highest goodness-of-fit, smallest number of descriptors and absence of collinearity between descriptors [37,38].

A complete randomization approach was applied to split of compounds in training (~2/3 compounds, 13 compounds), test (7 compounds: geranyl acetate, geranyl butyrate, geranyl tiglate, neral, neryl butyrate, neryl propanoate, citronellyl acetate, citronellyl propionate, and eugenol) and external (2 compounds: citronellyl acetate and neryl propanoate) sets.

Training set was used to identify the model, test set to validate the model and external set to assess the model external predictive power. The predictive power of identified models is sustained by an applied strategy; the models were not obtained on measured data which are subject of measurements errors. Instead, the QSAR models were constructed with population estimates (represented by Poisson parameter) that are less affected by errors. Thus, the QSAR models reflect the behavior of the compound on bacteria and fungi not the behavior of compound on a certain bacteria/fungus.

In order to assess the applicability domain of the obtained models, two approaches were involved on the full model with identified descriptors in the training sets [59]: leverage and identification of response outliers. A standardized measure of the distance between the descriptor values for the i^th^ observation and the means of the descriptor-values for all observations was computed to identify the leverage in descriptors (leverage value, *h*_i_). Whenever *h*_i_ > 3·(*k* + 1)/*n* (where *k* = number of independent variables in the model, *n* = sample size) compound was considered influential in the model [60] and was excluded from further analysis of the model. The response outliers were defined as compounds with absolute standardized residuals higher than 2.5. Leverage values (*h*_i_) *vs*. standardized residuals for compounds in training set was plotted to identify response outliers as well as independent variables with leverage values higher than threshold value (see Figures 2 and 3).

The model diagnostics was carried out using statistical parameters presented in Table 5.

The comparison of the models was performed using Steiger’s *Z* (association assumption between data) and Fisher’s *Z* (independence assumption of the data) statistics [68].

## 5. Conclusions

Antimicrobial activity of investigated oils, compounds and mixtures on the series of bacteria and fungi were shown to follow the Poisson distribution.

Two newly developed QSAR models, with Dragon and with SAPF descriptors, were found to be statistically significant internally. Even if the Dragon model proved to have higher goodness-of-fit, the model proved unacceptable in terms of prediction power. The SAPF model proved acceptable, with its prediction power being reliable, valid and stable in external validation analysis, with good overall performances in test set and test and external sets.

## Supplementary Information



## Figures and Tables

**Table 1:** Statistical parameters and population properties.

	λ	Mode	Mean	Var	StDev	Skew	EKurt	Median
**Compound (CID)**								
Citral (638011)	14.125	14	14.125	14.125	3.758	0.266	0.071	13.457
Geraniol (637566)	13.750	13	13.750	13.750	3.708	0.270	0.073	13.082
Geranyl formate (5282109)	8.875	8	8.875	8.875	2.979	0.336	0.113	8.207
Geranyl acetate (1549026)	8.200	8	8.200	8.200	2.864	0.349	0.122	7.531
Geranyl butyrate (5355856)	8.714	8	8.714	8.714	2.952	0.339	0.115	8.046
Geranyl tiglate (5367785)	11.625	11	11.625	11.625	3.410	0.293	0.086	10.957
Neral (643779)	13.500	13	13.500	13.500	3.674	0.272	0.074	12.932
Nerol (643820)	11.250	11	11.250	11.250	3.354	0.298	0.089	10.582
Nerol acetate (1549025)	7.333	7	7.333	7.333	2.708	0.369	0.136	6.664
Neryl butyrate (5352162)	10.714	10	10.714	10.714	3.273	0.306	0.093	10.046
Neryl propanoate (5365982)	10.714	10	10.714	10.714	3.273	0.306	0.093	10.046
Citronellal (7794)	14.600	14	14.600	14.600	3.821	0.262	0.068	13.932
Citronellyl formate (7778)	12.143	12	12.143	12.143	3.485	0.287	0.082	11.475
Citronellyl acetate (9017)	7.286	7	7.286	7.286	2.699	0.370	0.137	6.617
Citronellyl butyrate (8835)	8.167	8	8.167	8.167	2.858	0.350	0.122	7.498
Citronellyl isobutyrate (60985)	8.200	8	8.200	8.200	2.864	0.349	0.122	7.531
Citronellyl propionate (8834)	14.333	14	14.333	14.333	3.786	0.264	0.070	13.665
Hydroxycitronellal (7888)	18.750	18	18.750	18.750	4.330	0.231	0.053	18.083
Rose oxide (27866)	12.800	12	12.800	12.800	3.578	0.280	0.078	12.132
Eugenol (3314)	28.250	28	28.250	28.250	5.315	0.188	0.035	27.583
Sulfametrole (64939)	19.200	19	19.200	19.200	4.382	0.228	0.052	18.533
**Oil**								
Citronella	9.750	9	9.750	9.750	3.122	0.320	0.103	9.082
Geranium Africa	13.250	13	13.250	13.250	3.640	0.275	0.075	12.582
Geranium Bourbon	12.500	12	12.500	12.500	3.536	0.283	0.080	11.832
Geranium China	13.625	13	13.625	13.625	3.691	0.271	0.073	12.957
Helichrysum	10.667	10	10.667	10.667	3.266	0.306	0.094	9.999
Palmarosa	11.625	11	11.625	11.625	3.410	0.293	0.086	10.957
Rose	12.750	12	12.750	12.750	3.571	0.280	0.078	12.082
Verbena	16.500	16	16.500	16.500	4.062	0.246	0.061	15.833
**Mixture**								
Tetracycline hydrochloride	15.143	15	15.143	15.143	3.891	0.257	0.066	14.476
Ciproxin	26.000	26	26.000	26.000	5.099	0.196	0.038	25.333

λ = Parameter of Poisson distribution; Var = variance; StDev = standard deviation; Skew = skewness; EKurt = Excess Kurtosis.

**Table 2:** QSAR Residuals: Dragon *vs*. SAPF.

Set	CID	Y	Ŷ_Dragon_	Res_Dragon_	Ŷ_SAPF_	Res_SAPF_
Training	1549025	1.9924	2.0070	−0.0146	2.0761	−0.0836
Training	8835	2.1001	2.0564	0.0437	2.1461	−0.0460
Training	60985	2.1041	2.0768	0.0273	2.0553	0.0488
Training	5282109	2.1832	2.2596	−0.0764	2.3267	−0.1435
Training	643820	2.4204	2.6106	−0.1902	2.7127	−0.2923
Training	7778	2.4968	2.4132	0.0835	2.2816	0.2151
Training	27866	2.5494	2.5905	−0.0411	2.4957	0.0538
Training	637566	2.6210	2.6106	0.0104	2.7127	−0.0917
Training	638011	2.6479	2.7061	−0.0582	2.6042	0.0437
Training	8842	2.6741	2.6435	0.0307	2.5713	0.1029
Training	7794	2.6810	2.6929	−0.0118	2.6430	0.0380
Training	7888	2.9312	2.7346	0.1966	2.8638	0.0674
Training	64939	2.9549			2.8674	0.0875
Test	1549026	2.1041	2.0070	0.0971	2.2012	−0.0971
Test	5355856	2.1650	1.9271	0.2379	2.2830	−0.1180
Test	5352162	2.3716	1.9271	0.4445	2.7847	−0.4132
Test	5367785	2.4532	1.8661	0.5870	2.4642	−0.0111
Test	643779	2.6027	2.7061	−0.1034	2.6006	0.0021
Test	8834	2.6626	2.4108	0.2518	2.6207	0.0418
Test	3314	3.3411	2.7843	0.5568	3.3685	−0.0274
External	9017	1.9859	2.1432	−0.1572	2.0053	−0.0194
External	5365982	2.3716	2.2688	0.1028	2.2889	0.0827

CID = compound identification number; Y = observed ln(λ) value; Ŷ = estimated/predicted value; Res = residuals; Dragon = model from Equation(1); SAPF = model from Equation(2).

**Table 3:** Results of comparison: QSAR-Dragon model *vs*. QSAR-SAPF model.

Parameter (Abbreviation)	Dragon–Equation(1)–n = 21			SAPF–Equation(2)–n = 22		
Root-mean-square error (RMSE)	0.2314			0.1357		
Mean absolute error (MAE)	0.1582			0.0967		
Mean Absolute Percentage Error (MAPE)	0.0628			0.0403		
Standard error of prediction (SEP)	0.2371			0.0628		
Relative error of prediction (REP%)	9.2964			5.4523		
Predictive Power of the Model						
Q^2^_F_1__	0.2121 *			0.8436 *		
Q^2^_F_2__	0.2041 *			0.8421 *		
Q^2^_F_3__	n.a.			0.7742 *		
ρ_c-TR_	0.9457 a			0.9063 c		
ρ_c-TS_	0.4885 b			0.9219 d		
Fisher’s Predictive Power	TS	EX e	TS + EX f	TS	EX	TS + EX
*n*	7	2	9	7	2	9
*t*-value	3.1148	−0.2095	2.5071	−1.5344	0.6198	−1.2830
*p*-value	0.0104	0.4343	0.0230	0.0879	0.3234	0.1234

* = test set include also external compounds; ρ_c_ = concordance correlation coefficient; TR = training set; TS = test set;

a accuracy = 0.9985, precision = 0.9471;

b accuracy = 0.7357, precision = 0.6639;s

c accuracy = 0.9956, precision = 0.9103;

d accuracy = 0.9867, precision = 0.9344;

e = external set (two compounds);

f = training and external sets.

**Table 4:** Compounds, oils and mixtures: inhibition zones (mm).

		*SA*	*EF*	*EC*	*PV*	*PA*	*S*s	*KP*	*CA*	n
**Compound (CID)**										
1	Citral (638011)	15	23	11	9	10	8	9	28	8
2	Geraniol (637566)	15	12	15	12	11	10	10	25	8
3	Geranyl formate (5282109)	10	9	7	8	8	7	7	15	8
4	Geranyl acetate (1549026)	10	8	7	NIO	NIO	7	NIO	9	5
5	Geranyl butyrate (5355856)	10	11	7	NIO	9	7	7	10	7
6	Geranyl tiglate (5367785)	17	10	11	9	8	8	15	15	8
7	Neral (643779)	15	20	10	6	12	10	10	25	8
8	Nerol (643820)	11	8	10	10	10	7	7	27	8
9	Nerol acetate (1549025)	8	NIO	7	7	7	8	7	NIO	6
10	Neryl butyrate (5352162)	25	8	8	8	NIO	8	8	10	7
11	Neryl propanoate (5365982)	17	10	NIO	7	8	9	10	14	7
12	Citronellal (7794)	25	18	NIO	9	NIO	7	14	NIO	5
13	Citronellyl formate (7778)	18	20	10	8	9	7	NIO	13	7
14	Citronellyl acetate (9017)	10	6	NIO	6	7	6	7	9	7
15	Citronellyl butyrate (8835)	8	8	NIO	NIO	8	7	8	10	6
16	Citronellyl isobutyrate (60985)	8	10	9	7	NIO	NIO	7	NIO	5
17	Citronellyl propionate (8834)	15	20	NIO	NIO	10	15	11	15	6
18	Hydroxycitronellal (7888)	20	20	23	16	17	15	14	25	8
19	Rose oxide (27866)	8	10	NIO	11	7	NIO	NIO	28	5
20	Eugenol (3314)	30	30	28	28	25	25	28	32	8
21	Sulfametrole (64939)	27	27	11	23	NIO	8	NIO	NIO	5
32	Citronellol (8842)	25	18	NIO	8	NIO	7	NIO	NIO	4
**Oil**										
22	Citronella	10	10	7	10	7	7	7	20	8
23	Geranium Africa	16	12	10	10	10	9	11	28	8
24	Geranium Bourbon	13	12	8	12	10	10	10	25	8
25	Geranium China	20	13	14	9	9	9	10	25	8
26	Helichrysum	20	13	8	NIO	9	NIO	7	7	6
27	Palmarosa	8	13	12	9	11	10	10	20	8
28	Rose	20	15	10	10	8	9	10	20	8
29	Verbena	27	25	10	13	10	12	10	25	8
**Mixture**										
30	Tetracycline hydrochloride	15	22	11	13	15	10	20	NIO	7
31	Ciproxin	35	33	22	25	32	10	25	NIO	7

SA = *Staphylococcus aureus*; EF = *Enterococcus faecalis*; EC = *Escherichia coli*; PV = *Proteus vulgaris*; PA = *Pseudomonas aeruginosa*; SS = *Salmonella* sp.; KP = *Klebsiella pneumoniae*; CA = *Candida albicans; n* = sample size; NIO = No Inhibition Observed.

**Table 5:** Statistical parameters used to assess QSAR models.

Parameter (Abbreviation)	Formula [ref]	Remarks
Root-mean-square error (RMSE)	$\text{RMSE}=\sqrt{\frac{\sum_{\text{i}=1}^{\text{n}}{\left({\text{y}}_{\text{i}}-{\hat{\text{y}}}_{\text{i}}\right)}^{2}}{\text{n}}}$	RMSE > MAE → variation in the errors exist
Mean absolute error (MAE)	$\text{MAE}=\frac{\sum_{\text{i}=1}^{\text{n}}|{\text{y}}_{\text{i}}-{\hat{\text{y}}}_{\text{i}}|}{\text{n}}$	
Mean Absolute Percentage Error (MAPE) n	$\text{MAPE}=\frac{\sum_{\text{i}=1}^{\text{n}}|({\text{y}}_{\text{i}}-{\hat{\text{y}}}_{\text{i}})/{\text{y}}_{\text{i}}|}{\text{n}}$	MAPE ~ 0 → perfect fit
Standard error of prediction (SEP)	$\text{SEP}=\sqrt{\frac{\sum_{\text{i}=1}^{\text{n}}{\left({\hat{\text{y}}}_{\text{i}}-{\text{y}}_{\text{i}}\right)}^{2}}{\text{n}-1}}$	Lower value indicate a good model
Relative error of prediction (REP%)	$\text{REP(}\%\text{)}=\frac{100}{\bar{\text{y}}}\sqrt{\frac{\sum_{\text{i}=1}^{\text{n}}{\left({\hat{\text{y}}}_{\text{i}}-{\text{y}}_{\text{i}}\right)}^{2}}{\text{n}}}$	Lower value indicate a good model
Concordance analysis (ρ_c_)	$\rho_{\text{c}}=\frac{2\sum_{\text{i}=1}^{\text{n}}\left({\text{y}}_{\text{i}}-\bar{\text{y}}\right)\left({\hat{\text{y}}}_{\text{i}}-\bar{\hat{\text{y}}}\right)}{\sum_{\text{i}=1}^{\text{n}}{\left({\text{y}}_{\text{i}}-\bar{\text{y}}\right)}^{2}+\sum_{\text{i}=1}^{\text{n}}{\left({\hat{\text{y}}}_{\text{i}}-\bar{\hat{\text{y}}}\right)}^{2}+\text{n}{\left(\bar{\text{y}}-\bar{\hat{\text{y}}}\right)}^{2}}$ [61]	Strength of agreement [62]: >0.99 almost perfect; (0.95; 0.99) substantial; (0.90; 0.95) moderate; <0.90 poor
Predictive Power of the Model Prediction is considered accurate if the predictive power of the model is > 0.6 [66]	${\text{Q}}_{{\text{F}}_{1}}^{2}=1-\frac{\sum_{\text{i}=1}^{{\text{n}}_{\text{TS}}}{\left({\hat{\text{y}}}_{\text{i}}-{\text{y}}_{\text{i}}\right)}^{2}}{\sum_{\text{i}=1}^{{\text{n}}_{\text{TS}}}{\left({\text{y}}_{\text{i}}-{\bar{\text{y}}}_{\text{TR}}\right)}^{2}}$ [63]	Prediction power relative to mean value of observable in training set
	${\text{Q}}_{{\text{F}}_{2}}^{2}=1-\frac{\sum_{\text{i}=1}^{{\text{n}}_{\text{TS}}}{\left({\hat{\text{y}}}_{\text{i}}-{\text{y}}_{\text{i}}\right)}^{2}}{\sum_{\text{i}=1}^{{\text{n}}_{\text{TS}}}{\left({\text{y}}_{\text{i}}-{\bar{\text{y}}}_{\text{TS}}\right)}^{2}}$ [64]	Prediction power relative to mean value of observable in test set
	${\text{Q}}_{\text{F}3}^{2}=1-\frac{\left[\sum_{\text{i}=1}^{{\text{n}}_{\text{TS}}}{\left({\hat{\text{y}}}_{\text{i}}-{\text{y}}_{\text{i}}\right)}^{2}\right]/{\text{n}}_{\text{TS}}}{\left[\sum_{\text{i}=1}^{{\text{n}}_{\text{TS}}}{\left({\text{y}}_{\text{i}}-{\bar{\text{y}}}_{\text{TR}}\right)}^{2}\right]/{\text{n}}_{\text{TR}}}$ [65]	Overall prediction weighted by test set sample size relative to observable weighted by mean of observed value in training set weighted by sample size in training set
Predictive Power: Fisher’s approach	$\begin{array}{l} \text{t}=\frac{{\bar{\text{res}}}_{\text{TS}}-0}{\text{StDev}({\text{res}}_{\text{TS}})/\sqrt{{\text{n}}_{\text{TS}}}}\\ \text{p}=\text{TDIST}(\text{abs}(\text{t}),\;{\text{n}}_{\text{TS}}-1,1) \end{array}$ [67]	Evaluate if the mean of residual is statistically different by the expected value (0)

y_i_ = observed ln(λ) for i^th^ compound; ŷ_i_ = estimated/predicted ln(λ) by model from Equation(1), respectively Equation(2); *n* = sample size; ȳ = arithmetic mean of the observed ln(λ); $\bar{\hat{\text{y}}}$ = arithmetic mean of estimated/predicted ln(λ); ρ_c_ = concordance correlation coefficient; TR = training set; TS = test set; $\bar{\text{res}}$ = arithmetic mean of residuals; res = residuals; StDev = standard deviation; abs = absolute value.
