# Supplementary material for: Poisson Parameters of Antimicrobial Activity: A Quantitative Structure-Activity Approach
Source: Int J Mol Sci. 2012 Apr 24;13(4):5207–29. doi: 10.3390/ijms13045207 (PMC3344275; doi:10.3390/ijms13045207)
Supplement: Supplementary file 1 [file ijms-13-05207-s001.pdf]

Supplementary Information

Table. S.1. The analysis of probability distribution function for antimicrobial activity of a given chemical on bacteria series.

test	PubChem ID Compunds	SA	EF	EC	PV	PA	SS	KP	CA	N	pK-S Binomial	pA-D Binomial	pF-C-S Binomial	pK-S NegBino	pA-D NegBino	pF-C-S NegBino	pK-S Poisson	pA-D Poisson	pF-C-S Poisson	-ln(pC-S) Poisson	Poisson	Mode	Mean	Var	StDev	Skewness	EKurt	Median
Fit1	CID: 638011	15	23	11	9	10	8	9	28	8	0.0000	0.0000	0.0000	0.32	0.3153	0.3191	0.16	0.0446	0.0847	2.47	Poisson(14.125)	14	14.125	14.125	3.758	0.266	0.071	13.457
Fit2	CID: 637566	15	12	15	12	11	10	10	25	8	0.0000	0.0000	0.0000	0.49	0.4373	0.4631	0.65	0.4021	0.5129	0.67	Poisson(13.75)	13	13.750	13.750	3.708	0.270	0.073	13.082
Fit3	CID: 5282109	10	9	7	8	8	7	7	15	8	0.3432	0.3726	0.3576	0.00	0.0000	0.0000	0.25	0.3732	0.3077	1.18	Poisson(8.875)	8	8.875	8.875	2.979	0.336	0.113	8.207
Fit4	CID: 1549026	10	8	7			7		9	5	0.1851	0.0036	0.0258	0.00	0.0000	0.0000	0.25	0.3788	0.3055	1.19	Poisson(8.2)	8	8.200	8.200	2.864	0.349	0.122	7.531
Fit5	CID: 5355856	10	11	7		9	7	7	10	7	0.3654	0.2295	0.2896	0.00	0.0000	0.0000	0.26	0.3572	0.3052	1.19	Poisson(8.7143)	8	8.714	8.714	2.952	0.339	0.115	8.046
Fit6	CID: 5367785	17	10	11	9	8	8	15	15	8	0.5900	0.5226	0.5553	0.00	0.0000	0.0000	0.64	0.5493	0.5911	0.53	Poisson(11.625)	11	11.625	11.625	3.410	0.293	0.086	10.957
Fit7	CID: 643779	15	20	10	6	12	10	10	25	8	0.0000	0.0000	0.0000	0.38	0.4451	0.4138	0.44	0.2236	0.3125	1.16	Poisson(13.5)	13	13.500	13.500	3.674	0.272	0.074	12.932
Fit8	CID: 643820	11	8	10	10	10	7	7	27	8	0.0000	0.0000	0.0000	0.07	0.1390	0.0997	0.30	0.1231	0.1907	1.66	Poisson(11.25)	11	11.250	11.250	3.354	0.298	0.089	10.582
Fit9	CID: 1549025	8		7	7	7	8	7		6	0.0000	0.0000	0.0000	0.00	0.0000	0.0000	0.03	0.1072	0.0584	2.84	Poisson(7.3333)	7	7.333	7.333	2.708	0.369	0.136	6.664
Fit10	CID: 5352162	25	8	8	8		8	8	10	7	0.0000	0.0000	0.0000	0.02	0.0950	0.0430	0.08	0.0481	0.0603	2.81	Poisson(10.714)	10	10.714	10.714	3.273	0.306	0.093	10.046
Fit11	CID: 5365982	17	10		7	8	9	10	14	7	0.0000	0.0000	0.0000	0.83	0.6215	0.7164	0.82	0.6214	0.7146	0.34	Poisson(10.714)	10	10.714	10.714	3.273	0.306	0.093	10.046
Fit12	CID: 7794	25	18		9		7	14		5	0.0000	0.0000	0.0000	0.88	0.5941	0.7212	0.60	0.2271	0.3692	1.00	Poisson(14.6)	14	14.600	14.600	3.821	0.262	0.068	13.932
Fit13	CID: 7778	18	20	10	8	9	7		13	7	0.0000	0.0000	0.0000	0.86	0.5587	0.6917	0.71	0.3561	0.5043	0.68	Poisson(12.143)	12	12.143	12.143	3.485	0.287	0.082	11.475
Fit14	CID: 9017	10	6		6	7	6	7	9	7	0.2645	0.0333	0.0939	0.00	0.0000	0.0000	0.14	0.3259	0.2168	1.53	Poisson(7.2857)	7	7.286	7.286	2.699	0.370	0.137	6.617
Fit15	CID: 8835	8	8			8	7	8	10	6	0.1407	0.0043	0.0246	0.00	0.0000	0.0000	0.16	0.1734	0.1664	1.79	Poisson(8.1667)	8	8.167	8.167	2.858	0.350	0.122	7.498
Fit16	CID: 60985	8	10	9	7			7		5	0.1851	0.0036	0.0258	0.00	0.0000	0.0000	0.25	0.3788	0.3055	1.19	Poisson(8.2)	8	8.200	8.200	2.864	0.349	0.122	7.531
Fit17	CID: 8834	15	20			10	15	11	15	6	0.4467	0.5347	0.4898	0.00	0.0000	0.0000	0.54	0.5881	0.5654	0.57	Poisson(14.333)	14	14.333	14.333	3.786	0.264	0.070	13.665
Fit18	CID: 7888	20	20	23	16	17	15	14	25	8	0.8732	0.6737	0.7670	0.00	0.0000	0.0000	0.95	0.7175	0.8253	0.19	Poisson(18.75)	18	18.750	18.750	4.330	0.231	0.053	18.083
Fit19	CID: 27866	8	10		11	7			28	5	0.0000	0.0000	0.0000	0.41	0.4053	0.4101	0.24	0.0604	0.1215	2.11	Poisson(12.8)	12	12.800	12.800	3.578	0.280	0.078	12.132
Fit28	CID: 3314	30	30	28	28	25	25	28	32	8	0.1904	0.2398	0.2137	0.00	0.0000	0.0000	0.35	0.2878	0.3169	1.15	Poisson(28.25)	28	28.250	28.250	5.315	0.188	0.035	27.583
Fit30	CID: 64939	27	27	11	23		8			5	0.0000	0.0000	0.0000	0.27	0.3064	0.2862	0.22	0.0268	0.0767	2.57	Poisson(19.2)	19	19.200	19.200	4.382	0.228	0.052	18.533
																		F-C-S Poisson =		28.79	21	0.1190						
	Oils																											
Fit20	Citronella oil	10	10	7	10	7	7	7	20	8	0.0000	0.0000	0.0000	0.28	0.3097	0.2923	0.56	0.2565	0.3800	0.97	Poisson(9.75)	9	9.750	9.750	3.122	0.320	0.103	9.082
Fit21	Geranium oil Africa	16	12	10	10	10	9	11	28	8	0.0000	0.0000	0.0000	0.44	0.3325	0.3847	0.34	0.1554	0.2285	1.48	Poisson(13.25)	13	13.250	13.250	3.640	0.275	0.075	12.582
Fit22	Geranium oil Bourbon	13	12	8	12	10	10	10	25	8	0.0000	0.0000	0.0000	0.58	0.3612	0.4581	0.63	0.2883	0.4251	0.86	Poisson(12.5)	12	12.500	12.500	3.536	0.283	0.080	11.832
Fit23	Geranium oil China	20	13	14	9	9	9	10	25	8	0.0000	0.0000	0.0000	0.40	0.4409	0.4220	0.40	0.1967	0.2797	1.27	Poisson(13.625)	13	13.625	13.625	3.691	0.271	0.073	12.957
Fit24	Helichrysum oil	20	13	8		9		7	7	6	0.0000	0.0000	0.0000	0.56	0.5184	0.5376	0.60	0.3384	0.4517	0.79	Poisson(10.667)	10	10.667	10.667	3.266	0.306	0.094	9.999
Fit25	Palmarosa oil	8	13	12	9	11	10	10	20	8	0.0000	0.0000	0.0000	0.88	0.5334	0.6862	0.92	0.5378	0.7019	0.35	Poisson(11.625)	11	11.625	11.625	3.410	0.293	0.086	10.957
Fit26	Rose oil	20	15	10	10	8	9	10	20	8	0.0000	0.0000	0.0000	0.50	0.4157	0.4543	0.22	0.2273	0.2227	1.50	Poisson(12.75)	12	12.750	12.750	3.571	0.280	0.078	12.082
Fit27	Verbena oil	27	25	10	13	10	12	10	25	8	0.0000	0.0000	0.0000	0.39	0.2674	0.3242	0.13	0.0112	0.0387	3.25	Poisson(16.5)	16	16.500	16.500	4.062	0.246	0.061	15.833
																		F-C-S Poisson =		10.48	8	0.2332						
	Mixtures																											
Fit31	tetracycline hydrochloride	15	22	11	13	15	10	20		7	0.0000	0.0000	0.0000	0.93	0.6763	0.7916	0.90	0.6565	0.7707	0.26	Poisson(15.143)	15	15.143	15.143	3.891	0.257	0.066	14.476
Fit29	Ciproxin	35	33	22	25	32	10	25		7	0.0000	0.0000	0.0000	0.70	0.4299	0.5483	0.37	0.1719	0.2529	1.37	Poisson(26)	26	26.000	26.000	5.099	0.196	0.038	25.333
																				1.64	2	0.4415						
	Not enogh data																											
NoFit	CID: 8842	25	18		8		7			4											Poisson(14.5) new data	14	14.500	14.500	3.808	0.263	0.069	13.832

© 2012 by the authors; licensee MDPI, Basel, Switzerland. This article is an open access article distributed under the terms and conditions of the Creative Commons Attribution license (http://creativecommons.org/licenses/by/3.0/).
